# Lemierre's syndrome, reemergence of a forgotten disease: a case report

**DOI:** 10.1186/1757-1626-2-6397

**Published:** 2009-03-10

**Authors:** Anurag Kushawaha, Mohammed Popalzai, Eli El-Charabaty, Neville Mobarakai

**Affiliations:** 1 Department of Medicine, Staten Island University Hospital, 475 Seaview Avenue, Staten Island, NY 10305, USA

## Abstract

Lemierre's syndrome is a rare disorder of young adults caused by the anaerobic bacterium, *Fusobacterium necrophorum* and occasionally by other *Fusobacterium* species (*F. nucleatum, F. mortiferum and F. varium* etc). The condition is characterized by a primary oropharyngeal infection with evidence of septic thrombophlebitis, exhibited by positive blood cultures, clinical or radiographic evidence of internal jugular vein thrombosis, and at least one metastatic focus. The incidence of Lemierre's syndrome is reported to be nearly one in a million. In the pre-antibiotic era, Lemierre's syndrome followed a fulminant, often fatal course. During the 1960s and 1970s, the syndrome was rarely reported when penicillin was commonly prescribed to treat oropharyngeal infections. Today, antibiotic-resistant organisms are a major concern, thus causing more prudent prescribing of antibiotics. We present a case report of a 27-year-old man with worsening pharyngitis, which illustrates that subsequent reemergence of this often forgotten disease may become more common in clinical setting.

## Case presentation

A 27-year-old Caucasian male of Polish descent, with no prior medical history, was evaluated in the emergency department (ED) for a 2-day history of sore throat, nonproductive cough and fever. Physical exam revealed pharyngeal erythema and cervical lymphadenopathy. Monospot test was negative. Radiographic evaluation, blood cultures, and routine blood chemistries were obtained. He denied smoking, alcohol, illicit drug use or recent travel. He worked as a software programmer. He reported a penicillin allergy of a rash. At the time, the patient was diagnosed with pharyngitis and was discharged from the ED on oral azithromycin.

Twenty-four hours after the initial presentation, he was contacted at home for an abnormal chest x-ray consistent with right lower lobe pneumonia and advised to return to the ED for further evaluation. The patient now mentioned new-onset worsening dyspnea. Upon arrival in the ED, the patient was in severe respiratory distress. His respiratory rate was 40 breaths per minute, temperature was 101.2 °F, heart rate was 168 beats per minute, and blood pressure was 81/44 mmHg. Arterial blood gas analysis revealed pH of 7.50, PaCO_2_ of 26, and PaO_2_ of 52 on 100% oxygen non-rebreather mask. He was emergently intubated, fluid resuscitation and pressure support were initiated, and was admitted to the intensive care unit.

Preliminary blood work was notable for a WBC count was 24,000 cells/μL with differential count of 94% granulocytes. The initial chest radiograph now indicated new bilateral lung infiltrates consistent with adult respiratory distress syndrome (ARDS). Blood cultures were drawn and he was started on broad-spectrum antibiotics including intravenous linezolid, moxifloxacin, gentamicin, trimethoprim-sulfamethaxozole, cefepime, and metronidazole. Urine legionella antigen and legionella DFA were negative.

Forty-eight hours later, the initial gram stain of the blood indicated gram-negative rods from only the anaerobic vial. Interim blood cultures revealed *Fusobacterium varium*. The patient was desensitized to penicillin and the antibiotic regimen was changed to intravenous penicillin G and moxifloxacin. Computed tomography of the head and abdomen were unremarkable. Computed tomography of the chest revealed bilateral pulmonary nodules, moderate bilateral pleural effusions, bilateral infiltrates and complete collapse of the right lower lobe and left lower lobe. Computed tomography of the neck with contrast revealed lack of enhancement at the mid-portion of the left internal jugular vein. Doppler ultrasound of the neck revealed a left internal jugular vein thrombosis. Patient was diagnosed with Lemierre's syndrome. The patient was started on intravenous heparin. Antibiotics and anticoagulation were continued for several days. The patient continued to require ventilatory support for ARDS. The patient improved and was subsequently extubated 8 days later. He was discharged with an indwelling catheter on intravenous meropenem for two weeks, subcutaneous low-molecular-weight heparin for six weeks, and benadryl (penicillin allergy).

## Discussion

Fusobacteria are nonmotile, sporulating, obligate anaerobic Gram-negative rods that are normal flora in the human upper respiratory tract, gastrointestinal tract, and female genital tract. *Fusobacterium* exhibit classic tapered ends and filamentous forms, with or without swollen areas and large round bodies. Various toxins have been identified that are produced by Fusobacteria that may play a role in the pathogenicity. Unlike other anaerobic bacteria, Fusobacteria produce a lipopolysaccharide endotoxin with strong biologic activity, as well as a leukocidin and hemolysin, assisting in destruction of white and red blood cells[1]. Hemagglutinin production augments the fulminant nature of the disease, causing platelet aggregation and septic thrombus formation [2]. *Fusobacterium necrophorum and F nucleatum* are the species most often the causative agents of Lemierre's syndrome, but other *Fusobacterium* species can occasionally be found.

The exact mechanism of invasion and penetration of the pharyngeal mucosa has not been determined. Current hypothesis include the help of an underlying synergistic infectious process (bacterial or viral), with a concomitant decline in host resistance [3]. When pharyngitis due to *Fusobacterium* species occurs, the physical proximity of the vessels in the lateral pharyngeal space permits extension from the peritonsillar space to the internal jugular vein. This usually occurs in less than a week from the development of pharyngitis. Once invasion of the internal jugular vein is achieved, the resultant bacteremia triggers platelet aggregation and thrombus formation [4]. Thrombus formation and rapid bacterial growth result in a nidus for metastatic septic embolization.

The major clinical features of Lemierre's syndrome include primary infection of the oropharynx, bacteremia, radiographic or clinical evidence of internal jugular vein thrombosis, and septic metastatic foci. Accurate history and physical examination are needed for diagnosis. The syndrome should be suspected in young, otherwise healthy patients who have an underlying oropharyngeal infection and follow a worsening clinical course, requiring hospitalization for sepsis and worsening pulmonary symptoms in the setting of a recent pharyngeal infection. The patient's lateral neck swelling and tenderness (representing thrombophlebitis of the internal jugular or surrounding veins) is often mistaken for cervical lymphadenopathy. Patients can also demonstrate the "cord sign", an induration of the internal jugular vein beneath the anterior border of the sternocleidomastoid muscle [5]. Emboli from these veins metastasize to the pulmonary vasculature in up to 85% of patients, resulting in complicated pleural effusions, pulmonary abscesses, and Empyema [6]. The chest radiograph typically shows multiple nodular infiltrates scattered throughout both lung fields [7]. Adult respiratory distress syndrome (ARDS) occurs in a relatively small proportion of cases and fewer than 10% of cases reported in cited literature since 1990 have required mechanical ventilation. Other manifestations can include localized arthralgias and diffuse abdominal pain, which may represent septic embolic seeding of joints and abdominal microabscesses [8]. The hip is the most commonly infected joint in published series, but osteomyelitis in Lemierre's syndrome is rare. Abnormal coagulation studies and liver function tests, in the situation of hepatic seeding and abscess formation, may also be seen [9].

Imaging of the internal jugular vein and associated vasculature may be accomplished with ultrasound, computed tomography, magnetic resonance imaging to establish presence of thrombophlebitis [10]. Contrast computed tomography of the neck provides the definitive diagnosis, showing distended veins with enhancing walls, intraluminal filling defects, and swelling of adjacent soft tissues [11]. Ultrasonography can also confirm internal jugular vein thrombosis, showing localized echogenic regions within a dilated vessel [12]. Confirmation of Lemierre's syndrome is provided by demonstration of *Fusobacterium* species in anaerobic blood culture [13].

The recommended treatment of *Fusobacterium* species in Lemierre's syndrome is combination therapy with parenteral high dose penicillin and metronidazole. Intravenous clindamycin may be used in penicillin-allergic patients [14]. Pulmonary abscess and empyema must be addresses with definitive surgical drainage and evacuation. The role of anticoagulant therapy is controversial and is not presently recommended as a standard of care [15].

In conclusion, Lemierre's syndrome is a rare disorder caused by *Fusobacterium* species characterized by internal jugular vein thrombosis, oropharyngeal infection, septicemia, and presence of metastatic foci may be encountered today. The implications of this case are several and important. First, while many publications have discouraged the use of routine anaerobic blood cultures to guide antimicrobial therapy, this case illustrates the usefulness of having anaerobic bottles in blood culture sets. For a syndrome that is so characteristic, it is notable how often the diagnosis is missed until an anaerobic gram-negative rod is isolated from blood culture or other sterile site. There are several contributing factors for this. The variable manifestations of the septic emboli can distract clinicians from the initial oropharyngeal sepsis. In addition, the cases can present to a wide variety of specialties, including general medicine, otorhinolaryngology, pulmonology, orthopedics, and general surgery. The differential diagnosis of Lemierre's syndrome is vast and includes viral pharyngitis, infectious mononucleosis, acute retroviral syndrome, leptospirosis, acute bacterial pneumonia, atypical pneumonia, aspiration pneumonia, infective endocarditis, intra-abdominal sepsis.

Perhaps most importantly, most clinicians and even medical microbiologists have never seen a case. Although Lemierre's syndrome remains a rare disease, recent papers have suggested that the incidence of the condition is rising. More prudent and judicious antibiotic-prescribing habits in the setting of upper respiratory tract infections attributed to viral etiologies may lead to clinical reappearance of this often forgotten disease. Not only do antibiotics shorten the duration of symptoms related to bacterial pharyngitis, but can also prevent suppurative complications such as peritonsillar abscess. The typical clinical picture of Lemierre's syndrome is characteristic but many general practitioners are unaware of this condition and diagnosis is often delayed with potentially fatal complications. Physicians should be aware of this syndrome and should consider it in the differential diagnosis of a young patient suffering from a fulminant oropharyngeal infection with a deteriorating clinical course.

## Abbreviations

°F: degrees Fahrenheit; mmHg: millimeters of mercury; PaCO_2_: alveolar partial pressure of carbon dioxide; PaO_2_: alveolar partial pressure of oxygen; cells/ μL: cells per microliter; ARDS: adult respiratory distress syndrome; DFA: direct fluorescent antibody.

## Consent

"Written informed consent was obtained from the patient's next of kin/ family for publication of this case report. A copy of the written consent is available for review by the Editor-in-Chief of this journal."

## Competing interests

"The authors declare that they have no competing interests."

## Authors' contributions

AK conducted the literature review, interpreted the patient data, and was the major contributor to the manuscript. MP was involved in direct patient care during the hospitalization. EE was involved in direct patient care during the hospitalization as the hospitalist and was involved in manuscript revisions. NM was involved in direct patient care as the infectious disease consultant and was involved in manuscript revisions.
